# Exercise and COVID-19: exercise intensity reassures immunological benefits of post-COVID-19 condition

**DOI:** 10.3389/fphys.2023.1036925

**Published:** 2023-05-05

**Authors:** Anand Thirupathi, Wang Yong, Ofcan Oflaz, Eda Agascioglu, Yaodong Gu

**Affiliations:** ^1^ Research Academy of Medicine Combining Sports, Ningbo No 2 Hospital, Ningbo, China; ^2^ Faculty of Sports Science, Ningbo University, Ningbo, China; ^3^ Department of Medical Biology, Faculty of Medicine, Lokman Hekim University, Ankara, Türkiye; ^4^ Faculty of Sport Sciences, Yuksek Ihtisas University, Ankara, Türkiye

**Keywords:** COVID-19, SARS-CoV-2, exercise, intensity, duration, immune response

## Abstract

Any form of physical activity, including exercise, has various benefits at the physiological (improving cardiac and respiratory functions, increasing skeletal muscle mass, and maintaining homeostasis) and psychological levels (improving cognitive function, reducing anxiety and depression) which help to combat any type of infection. In contrast, the infectivity ratio could reduce the physical activity of an individual, such as performing a habitual exercise. Adaptation to different exercise strategies including intensity and duration may better increase physical performance and improve the symptoms. For example, low to moderate intensity perhaps fails to induce this adaptive process, while high-intensity of exercise compromises immune health. This can aggravate the infection rate (Open window theory). However, high intensity with a shorter time produces various morphological alterations in the primary organs including the lungs and heart, which facilitate life support in COVID-19 patients. However, less information about exercise protocols failed to assure the benefits of exercise to COVID-19 patients, particularly post-COVID-19 conditions. Therefore, this review will answer how exercise intensity is crucial to reassure the exercise benefits for promoting safe participation before infection and post-COVID-19 conditions.

## 1 Introduction

Although several pandemics have been successfully handled by the human community, the recent pandemic caused by the severe acute respiratory syndrome coronavirus-2 (SARS-CoV-2) has challenged the scientific community and put human life at risk (Hu et al., 2021). Although the initial outbreak was thought to be controllable, the increased number of SARS variants has aggravated the current challenge (Forni et al., 2021). According to the WHO, approximately 75 million people worldwide had been infected until the end of February 2023, including 0.6 million deaths (WHO coronavirus (COVID-19) dashboard); particularly, the mortality rate is higher in the elderly (Dadras et al., 2022; Watson et al., 2022). This may be due to compromised immune function, either in failure of immune functions or failure in the specific adaptive immune response, which is necessary for preventing the initial stage of infections (incubation period and asymptomatic conditions) (Chaplin, 2010; Walsh et al., 2011; Nicholson, 2016). Notably, after infection, the impact induced by COVID-19 is longer, so-called “long COVID-19,” which is often described with unnoticed symptoms such as fatigue, headache, and dyspnoea (Foged et al., 2021), and also alters immune systems. For example, muscle weakness and loss might be due to deconditioning that exists for several days due to local inflammation in the muscle which impaired the exercise response to COVID-19 (Fernandez-Gonzalo et al., 2014; Gjevestad et al., 2015; Rinaldo et al., 2021; Azzolino and Cesari, 2022). In addition, long COVID is known to impact all nervous system functions, and risk and recovery factors are poorly understood as of yet. Potentially; therefore, specific interventions such as exercise interventions, are necessary to boost longer-time immune function and nervous system functions. Maintaining physical fitness, proper nutrition, and good sleep improves immune function and nervous system functions.

Any type of physical activity, including exercise, is one of the factors in maintaining a healthy lifestyle (Rejeski et al., 2009). During this pandemic, due to COVID-19, sedentarism and physical inactivity (energy expenditure <1.5 metabolic equivalent of task (METs) during sitting, writing, and desk work) are increased (Park et al., 2020). This could compromise immune functions and decrease recovery and increase the infection rate, suggesting physical exercise is one of the most complementary tools in preventing COVID-19. However, results are mixed regarding exercise as an intervention for Long COVID, but the benefits of exercise are well-known. Despite mixed findings, there are some promising studies that have identified no adverse reactions (Mohamed and Alawna, 2021; McNarry et al., 2022). Previous studies reported that exercise could safeguard the host from other viral infections, such as influenza and herpes viruses (Martin et al., 2009; Domaszewska et al., 2022). A single bout of exercise (<60 min) can stimulate various immune cell types involved in immune surveillance and innate immune response (Nieman and Wentz, 2019; Campbell and Turner, 2018).

Exercise such as aerobic exercise and resistance exercise with different intensities improve immunoglobulin circulation and increase the anti-inflammatory cytokines, natural killer cells, and neutrophils which are all reshaping the immune function (Scheffer and Latini, 2020). Furthermore, moderate intensity in acute and chronic exercise decreases inflammation in the respiratory tract by augmenting immune defense (Philippou et al., 2012). Indeed, the immune system tries to keep homeostasis and triggers repair processes by coordinating the balance of pro and anti-inflammatory cytokines and signal molecules (Philippou et al., 2012). Chronic exercise decreases inflammation and prevents immune dysfunction in the elderly, suggesting the requirement of exercise for the elderly who are vulnerable to COVID-19 (da Silveira et al., 2021). However, exercise intensity also contributes to suppressing immune function. For example, less intensity with a longer duration could fail to induce adaptation (MacInnis and Gibala, 2017), while higher intensity induces immune suppression and allows various infections among the participants. Therefore, this review narrates the possible opportunity of finding how exercise remodifies the immune system to recommend exercise as the most potential lifestyle factor in managing COVID-19.

## 2 Exercise intensity in the remodifying immune system for COVID-19 management

Considering exercise intensity, higher exercise intensity perturbs the immune system (Table 1), as mentioned above, which possibly induces the “open window” theory explains that an acute bout of higher-intensity exercise suppresses the immune system (Peake et al., 2017). This may aggravate the COVID-19 infection possibly by post-exertional malaise. Therefore, exercise intensity is a crucial factor rather than type, duration, and individual capacity. In contrast, higher-intensity exercise restructures the major organs (Toti et al., 2013). This could efficiently manage long-COVID-19. For example, high-intensity exercise alters the morphology of the cardiac system and function in long-COVID-19 symptoms, possibly by improving glucose metabolism and reducing muscle wasting and inflammatory response (Rasmussen et al., 2009; Nigro et al., 2020; Rasmussen et al., 2021). In particular, high-intensity exercise aims at residual vascular inflammation, considered to be the major mechanism of long-COVID-19 (Christensen and Berg, 2021). Next, the high intensity may increase the adaptation in patients with various lung diseases. For example, high-intensity interval training (HIIT) for a short duration (within 2 weeks) elicits faster physiological adaptions than continuous moderate regular exercise by increasing interleukin-6 (IL-6) (Eaton et al., 2018). This HIIT-induced anti-inflammatory response increases the tolerance limit in patients with various diseases (Rahmati-Ahmadabad and Hosseini, 2020). However, this should be implemented after assessing the efficiency of the cardiovascular system, as higher-intensity exercise is linked with cardiac complications (Baird et al., 2017). However, this has been unproven by later studies (Eijsvogels et al., 2018), suggesting that high-intensity of exercise regimes, including HIIT, may be safe to perform during COVID-19. This may potentially save the time of the performer by increasing life. However, a combination of proper volume, exercise intensity, and types with optimum mode could also equally be a considerable factor. Particularly, it could be effective in asymptomatic and severe conditions. Nevertheless, studies reported that as long as the individual is well and asymptomatic, they are encouraged to perform moderate exercise rather than higher intensity exercise (Kim et al., 2021), which is evidenced by the performance of moderate exercise with 30–40 min weekly 3 to four times (Dixit, 2020). This can improve the immune system and possibly reduce viral infection. Short-term moderate-intensity exercise enhances the immune activity of tissue macrophages, increases the number of immunoglobulins, anti-inflammatory cytokines, neutrophils, NK cells, cytotoxic T, and immature B Cells, and enhances the participation of these components in the circulation. The exercise intensity, duration, ROS formation rate, and hormonal variations may also trigger the increase of leukocytes in the blood vessels and Pro - and anti-inflammatory cytokines levels (Wolach et al., 1998; Terra et al., 2012). Consequently, improves patients’ outcomes during infections before developing a serious threat (Wong et al., 2008; Martin et al., 2009). Moreover, short-term moderate exercise provides no high levels of proinflammatory cytokines. However, over time, exercise-induced immune control is triggered, thereby increasing selective lymphocyte subsets, resulting in reduced inflammation (Nie et al., 2021). In addition, studies have shown that HIIT improved immune surveillance by eliciting an anti-inflammatory response instead of a pro-inflammatory response in COVID-19 survivors (Wang et al., 2020; da Silveira et al., 2021). Regarding psychological management, high-intensity exercise improves mental health as moderate and low-intensity exercises, suggesting that high-intensity exercise with a shorter duration could be useful for efficient time management during quarantine (Olney et al., 2018; Nambi et al., 2022). Studies have shown that mild to moderate exercise decreases respiratory viral infection compared to higher-intensity exercise and sedentary conditions (Martin et al., 2009; Miles, 2009). However, this cannot be implemented at the initial stage of COVID-19 as it can aggravate the inflammatory response and further worsen the COVID-19 symptoms. Therefore, studies recommended refraining from performing an exercise to post-COVID-19 infection (Bhatia et al., 2020). This can be varied according to the expert’s opinion and individual activities. For example, people who are actively involved in some physical activities are asked to refrain from the exercise for 2 weeks after the symptoms are onset, and returning to performing exercise should be done gradually with no existing symptoms (Hughes et al., 2022).

**TABLE 1:** Effect of exercise on immune markers in COVID-19 condition.

Suitable markers for COVID-19 from immunological perspectives	Physical exercise influences the immunological markers in a dose-dependent manner	References
Tumor necrosis factor-alpha (TNF-alpha)	An increase of TNF-alpha can aggravate the severity of COVID-19	MacNeil et al. (2021); Guo et al. (2022)
Interleukin 17 (IL-17)	Targeting IL-17 can decrease the covid-1o	Sugama et al. (2013); Pacha et al. (2013)
Interferon gamma inducible protein-10 (IP-10) and Monocyte chemoattractant protein-1 (MCP-1)	Increase the COVID-19 disease severity	Docherty and Harley (2022); Chen and Wang (2020)
Macrophage inflammatory protein-1 alpha (MIP-1)	Risk factor of mortality in COVID-19	Middelbeek et al. (2021); Pons et al. (2021)
Type I interferons (IFN-I)	Decrease of IFN can severe the COVID-19	Buang et al. (2021); Palermo et al. (2021)
IL-4	IL-4 level is elevated for tissue remodeling in COVID-19	Conroy et al. (2016); Vaz de Paula et al. (2020)
IL-10	IL-10 level is linked with poor health and severity in COVID-19	Cabral-Santos et al. (2019); Lu et al. (2021)
IL-13	Elevated level of IL-13 is linked with the requirement of mechanical ventilation in COVID-19	Knudsen et al. (2020); Donlan et al. (2021)
CD4 and CD8	Increased level of CD4 and CD8 worsen the COVID-19	Bernal et al. (2021); Remy et al. (2020)

## 3 The link between duration and intensity in overcoming COVID-19

**FIGURE 1:**
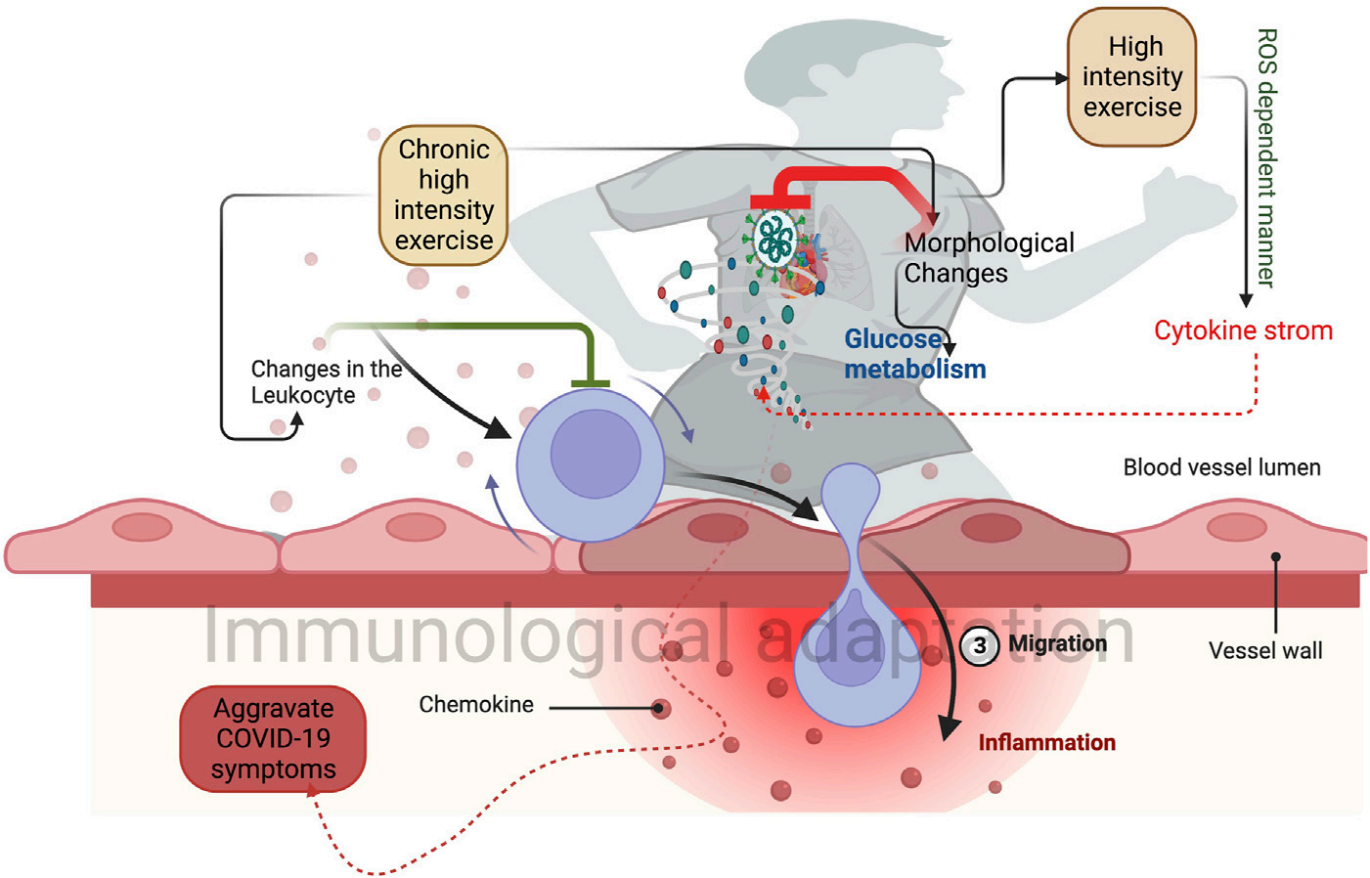
Higher-intensity exercise induces morphological changes in the lungs and heart. This can improve glucose metabolism and reduce the post covid symptoms. Chronic exercise-induced changes in the leukocytes prevent the proinflammatory response (Green inhibitory mark) and induce immunological adaptation. Sudden exercise in a shorter time induces a group of cytokine storms that prevent the SARS-CoV-2 infection.

Although lower to moderate intensity exercise with longer duration perhaps provides benefits, it is arguable how and at what point the adaptive signaling is to be activated. Also, it is difficult to obtain the required physiological limit while performing moderate exercise. Exercise with higher intensity produces required morphological changes in all the tissues, including the lungs and heart (Figure 1). This may help to sustain COVID-19 post-symptoms, as discussed previously. Next, short-duration exercise provides physiological adaptation in the high intensity than the moderate with less recovery time. However, how this adaptative response is varied from higher to moderate intensity needs further investigation. For instance, shorter duration with higher intensity activates various adaptive response signaling targets such as the 5′AMP-activated protein kinase (AMPK), peroxisome proliferator-activated receptor-gamma coactivator (PGC-1 alpha), and ROS pathways. Further, lactate increases ROS formation, which seems responsible for lactate-induced cellular adaptations to higher-intensity exercises (Nalbandian and Takeda, 2016; Dankel et al., 2017). Lactate increases PGC1-alpha expression in C2C12 myotubes in a concentration-dependent manner, which directly represents exercise intensity. Lactate mediates the PGC1 alpha upregulation via the formation of ROS (Nalbandian et al., 2019). A study reported the elevated level of lactate dehydrogenase in survivors and non-survivors of severe COVID-19 illness (Zhou et al., 2020), suggesting the role of lactate as an important indicator of prescribing and designing exercise for COVID-19 patients. However, how this adaptive signaling is helpful to COVID-19 alleviation needs further investigation. Furthermore, ROS cascade products such as hydrogen peroxide (H2O2), which acts as an important signaling molecule, are required for PGC-1 alpha upregulation in contracting skeletal muscle cells (Barbosa et al., 2013). Moreover, H2O2 reduces cellular ATP levels, activating AMPK, and increases the PGC-1 alpha mRNA level (Irrcher et al., 2003). In other words, H2O2 can promote PGC-1 alpha expression via AMPK. Indeed, PGC-1 alpha expression is related to ROS level, which seems to be dependent on exercise intensity-related lactate formation (Radak et al., 2013; Radak et al., 2017). Lactate and ROS can be formed in all types of exercise. However, ROS might illustrate toxic effects on cellular compartments during high-intensity exercise with a longer duration, not a shorter duration. It might be possible to eliminate over-formed ROS during high-intensity exercise in a short duration like HIIT due to having a chance of more oxygenation in intervals periods of exercise. On the other hand, high-intensity exercise for a long duration (i.e., sustained anaerobic metabolism), leads to lactate increase and metabolic acidosis, which would mean physiologically over-formed ROS and suppressed immune functions.

**FIGURE 2:**
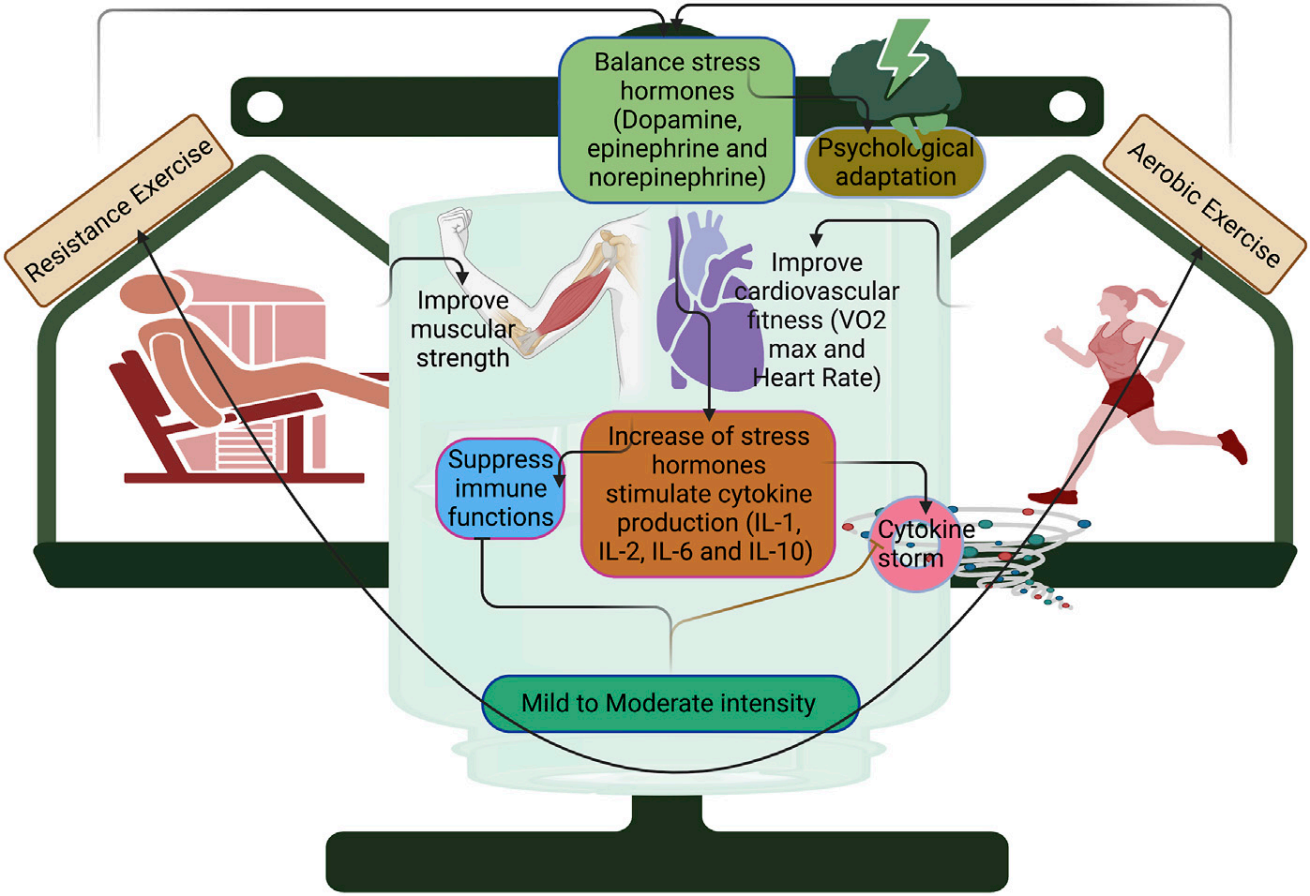
Resistance and aerobic training with mild and moderate intensity improve cardiovascular fitness and muscular strength in COVID-19. Mild and moderate intensity can balance the stress hormones such as dopamine, epinephrine, and norepinephrine for producing psychological adaptation, otherwise; it can stimulate cytokine production and suppress immune functions.

Overall, cellular adaptation and immune response to high-intensity exercise in a short duration would be better with higher endogenic antioxidant activities. Despite these, the clinical characteristics and fitness level of an individual are crucial criteria to determine which exercise to do and its efficacy, including COVID-19, which could disturb metabolic health, and in turn, worsen the infection rate in COVID-19. Studies have reported that metabolic abnormalities are observed in SARS-COV-2 infection (Wu et al., 2017; Ayres, 2020), suggesting the need for moderate exercise rather than higher-intensity exercise. Moderate exercise with 20–30 min (treadmill running) reduced the infection rate and mortality. Regarding higher intensity with longer duration, the J-shape link has been implemented with the performer of exercise protocols consisting of higher intensity or longer periods that allow susceptible to URT1, proposing higher intensity exercise requires shorter duration during COVID-19. However, successful immunomodulation against COVID-19 depends on the interaction between intensity and duration. Moderate intensity (50%–70% VO2 max) 3 times per week may increase the immune function through oxidative burst and cytolytic activity of NK cells (Deng et al., 2022). In COVID-19, moderate exercise improved the NK cell functions and reduced the proinflammatory cytokines (van Eeden et al., 2020). Moreover, moderate exercise leads to the downregulation of Toll-like receptors and/or cytokines, which results in an anti-inflammatory effect (Rodriguez-Miguelez et al., 2014; Suzuki et al., 2021). In contrast, chronic endurance exercise with high intensity could induce transcriptional changes in the peripheral system, including leukocytes and increase the expression of genes related to mitochondrial energetics, and downregulate the pro-inflammatory genes (Liu et al., 2017), while continuous prolonged exercise with higher intensity increases the inflammatory response (>2 h) than shorter duration (approximately 20 min). Chronic endurance exercise with high intensity could lead to reduced expression of Toll-like receptors in macrophages and the presentation of antigens to T lymphocytes, which in turn diminishes the inflammatory T helper type 1 response, and this might cause chronic inflammation (Gleeson et al., 2006). IL-10 is produced by the innate and cellular immune systems, called cytokine synthesis inhibitory factors, due to long-term high-intensity exercise (Moore et al., 2001). Furthermore, stress hormones like cortisol and catecholamines might lead to immune suppression during high-intensity exercise (Suzuki et al., 2000). For example, Gubbi et al. (2020) reported a balance of catecholamines in COVID-19 patients regulates immune functions, otherwise; it can stimulate various components of the immune system such as IL-1, IL-2, IL-6, and IL-10 (Figure 2). However, this should be established with COVID-19 patients before and after infection with different types of exercises. Three days a week of resistance (50% 1RM [one-repetition maximum]) and aerobic exercise interventions (30–60 min, 65%–70% heart rate reserve (HRR)) improve cardiovascular fitness and muscular strength in mild COVID-19 patients, but this study did not verify the exercise stress before of infection (Jimeno-Almazán et al., 2022). Assessing the exercise that caused stress before the infection and post-COVID-19 condition may better formulate the exercise protocols for quicker recovery from longer COVID-19 symptoms.

## 4 Future perspectives

Exercise with mild and moderate intensity can play a major role in recovering an active lifestyle in post-COVID-19 conditions. This may be due to the improvement of cardiovascular fitness and skeletal muscle strength in COVID-19 conditions, especially in the later stages of COVID-19 conditions including suffering from long-term disability. However, extrapolating the available data with combinations of other exercise protocols such as high-intensity interval training can give different health outcomes in COVID-19 conditions including improving neurocognitive and behavioral strategies.

## 5 Search strategy and selection criteria

The literature review was done on Pubmed, Web of Science, and Google Scholar using search terms “COVID-19, SARC-COV-2, immune system, resistance training, and aerobic training in combination with the term exercise. Data were reviewed by the two internal reviewers from the full-length articles, such as case reports, observational retrospective studies, systematic reviews, and meta-analyses published from 2020 to April 2023. Studies that were not related to exercise and COVID-19 were excluded from this study.

## 6 Conclusion

Low to moderate resistance and endurance training is a more effective and well-tolerated intervention in post-COVID-19. However, this scenario may fail to activate various adaptive signalings that are required for increasing physiological limits and further morphological alterations in major organs including lungs and heart. In addition, exercise intensity influences the levels of stress hormones such as catecholamines (adrenaline, noradrenaline, and dopamine) which are more closely linked with clinical characteristics of COVID-19 including metabolic alterations and immune dysregulation. This may severe the COVID-19 symptoms. For example, exercise-induced catecholamines can stimulate the number of immune components such as increasing cytokine production (IL-1, IL-2, IL-6) and suppressing T-lymphocyte production. Furthermore, these stress hormones can produce multiple effects on psychological adaptation. Therefore, future studies in setting up a precise exercise intensity with different types can warrant COVID-19 patients to overcome all these clinical characteristics and improve the patient’s life quality.
